# Association of attachment with level of physical activity among dog owners: A cross-sectional study

**DOI:** 10.1371/journal.pone.0313160

**Published:** 2024-11-27

**Authors:** Yu Taniguchi, Tomoko Ikeuchi

**Affiliations:** 1 National Institute for Environmental Studies, Japan Environment and Children’s Study Programme Office, Ibaraki, Japan; 2 Research on Social and Human Sciences, Tokyo Metropolitan Institute for Geriatrics and Gerontology, Tokyo, Japan; Mansheyet El Bakry General Hospital, EGYPT

## Abstract

This cross-sectional study examined the association of attachment with the frequency of dog walking and physical activity level among dog owners. We analyzed data collected in an internet survey conducted by the Japan Pet Food Association in 2023. Valid data were obtained from 1041 dog owners. The mean (SD) age of participants was 52.5 (14.9) years, with 57.5% being women. Ordinal logistic regression models showed that a higher attachment score was associated with a higher frequency of dog walking after controlling for important confounders (B = 0.04, SE = 0.02, p<0.01, Adjusted R^2^ = 0.03). Linear regression models showed that a higher attachment score was associated with a higher moderate-vigorous physical activity level (B = 1.43, SE = 0.44, p<0.01, Adjusted R^2^ = 0.01). These results suggest that dog owners with strong attachment maintain physical activity levels through caring for their dogs. Higher levels of physical activity might have protective effects against adverse health outcomes.

## 1. Introduction

Studies over the last few decades on human–animal interaction have reported that dog ownership is associated with positive effects on blood pressure response [1], cardiovascular risk [2,3], ADL level [4], frailty risk [5,6], dementia risk [7], disability risk [8], and mortality risk [3,8–10]. It is well known that low physical activity levels are a risk factor for these adverse health outcomes. Previous evidence from a review reported that dog owners of all ages, including older adults, take more exercise than non-owners [11].

Based on this accumulated evidence, we previously examined the association of interactions between dog ownership and exercise habits with dementia [7] and disability [8], and revealed that dog owners with a regular exercise habit had lower risks for adverse health outcomes. One of our studies [7] also showed that approximately 20% of dog owners do not have a regular exercise habit, indicating that even dog owners do not necessarily have this habit. The second study reported similar findings [8].

The reason for these differences in exercise habit among dog owners is not clear. Pet attachment has recently attracted research interest [12–14] on human–animal interaction, however, previous studies of pet attachment examined associations with family climate; loneliness and depressed mood; and perceived stress and life satisfaction. To our knowledge, the association of attachment with physical activity has received little attention, with only one study reported to date among 38 dog owners using an ActiGraph wGT3X-Link accelerometer [15]. Further, no epidemiological studies have examined the association with exclusion of the influence of confounding factors. We hypothesized that the strength of the attachment of dog owners may affect their physical activity level through daily care for their dogs.

Here, to help identify important factors between dog ownership and adverse health outcomes, we conducted a cross-sectional epidemiological study to examine the association of attachment with the frequency of dog walking and physical activity level among dog owners.

## 2. Methods

### 2.1. Participants

We used data collected in a survey conducted by the Japan Pet Food Association in 2023. Briefly, the Japan Pet Food Association has conducted annual internet surveys since 2004 to determine the number of animals in each household and the conditions of their living environment. In October 2023, the Association commissioned an internet survey to a research company which surveyed individuals aged 20 to 79 years who were sampled from all over Japan by place of residence and confirmed their willingness to participate. The survey included 1683 owners of dogs, cats, or both.

Informed consent was obtained via the website from all participants before the survey was conducted, and all protocols were approved by the Ethics Committee of the National Institute for Environmental Studies. We adhered strictly to the Declaration of Helsinki. The authors were unable to access information that could identify individual participants during or after data collection.

### 2.2. Definition of pet attachment

We assessed psychometric properties using the shortened version of an existing attachment measure, the CENSHARE Pet Attachment Survey [16]. This scale had 27 questions originally [17] of which 6 were selected for the shortened version. Because the shortened version was in English, we conducted a back-translation between English and Japanese (S1 Fig). A previous study used this brief scale among children in a wide range of ages and their primary caregivers [16] and reported that the response distribution to the 6 questions showed similar values to a previous study [16], and that reliability was high (Table 1). For analysis, “almost always” was coded as 4 and “never” as 1. Attachment score calculated using the 6 items had a maximum score of 24 [16], with higher scores indicating higher pet attachment among dog owners.

**Table 1:** Distribution of responses to the pet attachment questionnaire and its internal consistency among dog owners.

	Never (%)	Sometimes (%)	Often (%)	Almost always (%)	Correlation with total score
Q1. How often do you spend time each day playing with or exercising your pet?	2.6	18.6	32.2	46.6	0.628
Q2. How often is your pet aware of your different moods?	13.3	30.2	31.9	24.7	0.695
Q3. When you come home, how often is your pet the first one you greet?	5.7	10.9	24.0	59.5	0.683
Q4. When you feel bad, how often do you seek your pet for comfort?	13.6	28.3	31.7	26.3	0.687
Q5. How often do you consider your pet to be a member of your family?	1.9	7.4	16.1	74.5	0.633
Q6. How often do you have your pet near you when you study, read, or watch TV?	8.6	14.3	31.1	45.9	0.710
Total reliability (Cronbach Alpha)					0.756

### 2.3. Other measurements

The covariates included demographic characteristics such as sex, age, living alone, number of children living together, marital status, housing type, income, frequency of dog walking, moderate-vigorous physical activity per week, dog size, monthly pet-related expenses, and number of pets kept in the past 10 years. Frequency of dog walking was categorized into the 4 groups of 2 or more times a day, 1–2 times a day, 3–7 times a week, or 3 or fewer times a week. Moderate-vigorous physical activity was assessed using the International Physical Activity Questionnaire Short-Form [18] and is shown as METs-hours/week.

### 2.4. Statistical analyses

The primary statistical technique used to analyze the survey data was ordinal logistic regression and linear regression. Ordinal logistic regression was applied to examine the association of attachment with frequency of dog walking, because frequency of dog walking is categorical data. To select a model to examine the association of attachment with level of physical activity, we used curve estimation, and then analyzed the linear regression of attachment with physical activity level, because physical activity level is quantitative data. Potential confounders were evaluated for collinearity and included sex, age, living alone, marital status, housing type, and income. Statistical analyses were done with SPSS (version 23.0; SPSS, Inc., Chicago, IL, USA). P values of less than .05 were considered statistically significant.

## 3. Results

### 3.1. Characteristics and pet attachment

Among 1683 dog and/or cat owners, 1041 were dog owners, more than half of whom had very small dogs (Table 2). The mean (SD) age of participants was 52.5 (14.9) years, and 57.5% were women. 10.4% were living alone and 89.6% were living together. 58.8% did not live with children and 71.1% were married. 70.0% owned a house and 10.5% lived in an owned apartment. Median income was 5 million—6 million yen.

**Table 2:** Demographic characteristics among dog owners.

Variable	Dog owners (n = 1041)
Sex (female %)	57.5
Age, mean years (SD)	52.5 (14.9)
Living alone (%)	10.4
Number of children living together (%)	
4 or more	1.0
3 people	3.4
2 people	14.3
1 person	22.6
none	58.8
Marital status (married %)	71.1
Housing type (%)	
owned house	70.0
owned apartment	10.5
rented house	3.1
rented apartment	14.7
other	1.7
Income (%)	
≥ 20 million yen	2.3
15 million—20 million yen	2.4
12 million—15 million yen	4.4
10 million—12 million yen	4.9
9 million—10 million yen	7.4
8 million—9 million yen	3.8
7 million—8 million yen	7.5
6 million—7 million yen	8.3
5 million—6 million yen	11.5
4 million—5 million yen	12.6
3 million—4 million yen	12.4
2 million—3 million yen	12.3
1 million—2 million yen	5.0
< 1 million yen	4.8
Frequency of dog walking (%)	
2 or more times a day	25.1
1–2 times a day	3.8
3–7 times a week	45.8
3 or fewer times a week	25.3
Moderate-vigorous physical activity per week, mean METs-hours/week (SD)	41.4 (50.5)
Dog size (%)	
middle-large	12.3
small	33.4
very small	53.7
unknown	0.6
Monthly pet-related expenses, mean yen (SD)	17776 (17870)
Number of pets kept in the past 10 years, mean (SD)	1.7 (1.3)
Pet attachment, mean score (SD)	18.8 (3.6)

SD: Standard Deviation.

The mean (SD) pet attachment score was 18.8 (3.6) and minimum and maximum scores were 6 and 24. Regarding dog walking, frequency was 25.1% for 2 or more times a day, 3.8% for 1–2 times a day, 45.8% for 3–7 times a week, and 25.3% for 3 or fewer times a week. The mean (SD) moderate-vigorous physical activity was 41.4 (50.5) METs-hours/week and minimum and maximum values were 0 and 498.

### 3.2. Association of higher attachment with frequency of dog walking and physical activity level

Ordinal logistic regression models showed that a higher attachment score was associated with a higher frequency of dog walking in Model-1 (B = 0.04, SE = 0.02, p<0.01) (Table 3). Closely similar results were obtained in the model which fully adjusted for sex, age, living alone, marital status, housing type, and income (B = 0.04, SE = 0.02, p<0.01). Linear regression models showed that a higher attachment score was associated with a higher moderate-vigorous physical activity level in Model-1 (B = 1.37, SE = 0.44, p<0.01). These results were closely similar in the fully adjusted model (B = 1.43, SE = 0.44, p<0.01).

**Table 3:** Association of attachment with frequency of dog walking and level of physical activity among dog owners.

	Dependent variable							
	Frequency of dog walking				Level of physical activity			
	B (SE)	95% CI	p-value	Adjusted R^2^	B (SE)	95% CI	p-value	Adjusted R^2^
Independent variable								
Pet attachment score: Model 1	0.04 (0.02)	0.01–0.07	< 0.01	0.019	1.37 (0.44)	0.52–2.23	< 0.01	0.011
Pet attachment score: Model 2	0.04 (0.02)	0.01–0.07	< 0.01	0.033	1.43 (0.44)	0.57–2.28	< 0.01	0.010

SE: Standard error. CI: Confidence interval.

Ordinal logistic regression was calculated for frequency of dog walking, and linear regression was calculated for level of physical activity.

Model 1: Adjusted for sex and age.

Model 2: Adjusted for model 1 plus living alone, marital status, housing type, and income.

## 4. Discussion

The main findings of this cross-sectional epidemiological study were that higher attachment is associated with higher frequency of dog walking, and that dog owners have a higher physical activity level after controlling for demographic characteristics.

Westgarth et al reported that dog ownership is associated with more recreational walking and considerably greater odds of meeting PA guidelines [19]. Although other studies have supported this evidence [11,19–22], dog owners do not necessarily have an exercise habit or higher physical activity level. Our present study showed that the strength of attachment is an important factor in dog walking and physical activity levels among dog owners. As a mechanism underlying dog ownership and health benefits, dog owners with strong attachment maintain their physical activity level through caring for their dogs. Higher levels of physical activity might have protective effects on cardiovascular risk [2,3], ADL level [4], frailty risk [5,6], cognitive function [23–25], dementia risk [7], and disability risk [8], and thereby ultimately contribute to lowering mortality risk [3,8,9,26,27].

Moreover, the study also showed that even among dog owners, weak attachment was associated with lower dog walking frequency and lower physical activity levels. This is important evidence suggesting that the simple fact of dog ownership alone does not confer health benefits. Furthermore, if dog owners have a weak attachment, they will have less frequency of dog walking, which suggests that the dog’s physical activity level will also be lower. This may suggest that the strength of attachment may affect the health status of both the owner and their dog. The results of this study will contribute to identifying the targets who may benefit from health promotion policies based on the findings of human-animal interaction.

This epidemiological study has some strengths that warrant mention. First, our sample enabled the control of confounding factors such as demographic characteristics in examining the independent associations of attachment with both the frequency of dog walking and physical activity level. Next, we created a shortened version of an attachment measure in Japanese. Although it is still necessary to confirm the concurrent validity of this Japanese version of the scale, its reliability was confirmed in this study (Cronbach Alpha = 0.756), indicating its usefulness for future research on human–animal interaction in Japan. Third, the data source in this study was based on the internet surveys to determine the number of animals in each household and the conditions of their living environment. The results of this study may be considered generalizable to Japanese dog owners.

Nevertheless, this study also had a number of limitations. First, although we examined the direct effect of attachment on physical activity, indirect mechanisms such as psychological [28] and social effects [29] on physical activity are also possible. Krause-Parello et al. reported that pet attachment support, but not human social support, influenced the relationship between loneliness and depressed mood, indicating the importance of pet attachment as a greater form of support in this sample [13]. Future research should investigate indirect effects between attachment strength and physical activity in epidemiological study for adverse health outcomes. Second, it has been reported that strong attachment has negative health effects, such as higher attachment to their pets predicted depression [29,30]. Although this study found that higher attachment had a positive effect on physical activity, the risks and benefits of strong attachment on health outcomes warrants additional research focus. Third, owing to its cross-sectional design, this study cannot clarify the causal relationship between attachment and dog ownership. Fourth, this study did not collect the information of dog size or strain. We speculate that larger and more active dogs are presumably given more walks than smaller and less active breeds. Fifth, we assessed pet attachment with an existing attachment measure after a back-translation between English and Japanese. Although we confirmed Cronbach’s Alpha of its score in this study, future study is needed to confirm its validity. Finally, the data were collected through an internet survey, and a degree of selection bias related to participant ability to respond to an internet survey may have been present. In this study, the association between the attachment of dog owners and their physical activity level in subjects aged 80 and older is unclear.

In summary, this cross-sectional study is to our knowledge the first study to show that higher attachment is associated with a higher frequency of dog walking and higher physical activity level among dog owners after controlling for demographic characteristics among Japanese dog owners. Dog owners with a strong attachment maintain physical activity levels through caring for their dogs, and higher levels of physical activity might have protective effects against adverse health outcomes. Dog owners with a strong attachment may have benefited most from health promotion policies based on the findings of human-animal interaction.

## Supporting information

S1 Fig Questionnaire on pet attachment in Japanese. (DOCX)
