# Supplementary material for: Association of attachment with level of physical activity among dog owners: A cross-sectional study
Source: PLoS One. 2024 Nov 27;19(11):e0313160. doi: 10.1371/journal.pone.0313160 (PMC11602116; doi:10.1371/journal.pone.0313160)
Supplement: S1 Fig — (DOCX) [file pone.0313160.s001.docx]

Supplemental Figure 1. Questionnaire on pet attachment in Japanese.


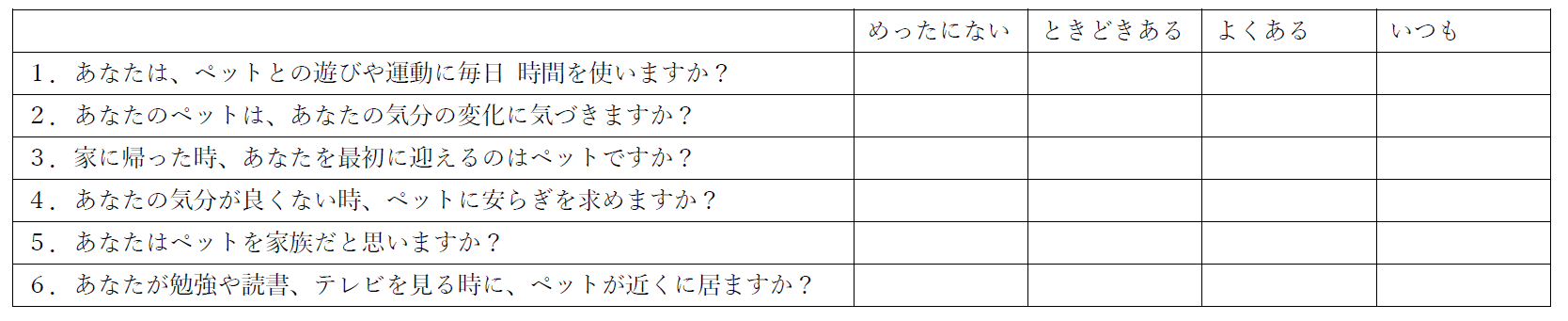


1. How often do you spend time each day playing with or exercising your pet?
2. How often is your pet aware of your different moods?
3. When you come home, how often is your pet the first one you greet?
4. When you feel bad, how often do you seek your pet for comfort?
5. How often do you consider your pet to be a member of your family?
6. How often do you have your pet near you when you study, read, or watch TV?
